# Effect of closed reduction and external fixation in elderly patients with anterior shoulder dislocation combined with humeral surgical neck fracture: a retrospective cohort study based on fracture-dislocation classification

**DOI:** 10.3389/fsurg.2026.1853922

**Published:** 2026-09-10

**Authors:** Feng Dai, Lei Dong, Songyi Qiao, Taotao Song, Zhen Lu, Xueqiang Shen

**Affiliations:** 1 Department of Orthopedic Surgery, Suzhou TCM Hospital Affiliated to Nanjing University of Chinese Medicine, Suzhou, Jiangsu, China; 2 Department of Orthopedic Surgery, Huaian Hospital of Huaian City, Huaian, Jiangsu, China; 3 Department of Orthopedic Surgery, Jinshazhou Hospital Affiliated to Guangzhou University of Chinese Medicine, Guangzhou, Guangdong, China; 4 Department of Traditional Chinese Medicine, Wujiang District Pingwang Community Health Service Center, Suzhou, Jiangsu, China

**Keywords:** anterior shoulder dislocation, closed reduction, elderly patients, external fixation, fracture-dislocation classification, humeral surgical neck fracture

## Abstract

**Background:**

Anterior shoulder dislocation combined with humeral surgical neck fracture is a severe injury that often requires emergency surgery. However, for some patients, such as those of advanced age or with surgical contraindications, closed reduction can be considered. This retrospective cohort study aimed to confirm the feasibility and clinical outcomes of the closed reduction technique with effective external fixation for the treatment of anterior shoulder dislocation combined with humeral surgical neck fracture in elderly patients.

**Method:**

From January 2010 to March 2024, a total of 51 patients with anterior shoulder dislocation combined with humeral surgical neck fracture from Suzhou TCM Hospital Affiliated to Nanjing University of Chinese Medicine participated in this study. All patients underwent emergency closed reduction and external fixation. Whether they would be finally included in the statistical analysis was determined based on the post-reduction imaging results. We conducted follow-up for more than one year on the patients who were finally included in the study. We also recorded patients' general characteristics, fracture-dislocation classification (type I, type II, type III), fracture reduction and healing status, as well as the affected limb's function scores.

**Result:**

After screening, 38 patients were included in the cohort, and ultimately 34 patients completed the follow-up. Among them, there were 11 males and 23 females. Their ages ranged from 65 to 90 years, with an average of (74.15 ± 7.36) years. All 34 patients comprised 15 cases of type I, 14 cases of type II, and 5 cases of type III, respectively. Notably, 64.71% (22/34) of them achieved successful closed reduction, including 13 cases of type I, 9 cases of type II, and no case of type III. At the last follow-up, 8.82% (3/34) and 5.88% (2/34) of patients experienced humeral head ischemic necrosis and nonunion of fractures, respectively. The Constant Murley shoulder function score for conservative treatment patients (*n* = 21) ranged from 68 to 95 points, with an average of (83.90 ± 6.62) points.

**Conclusion:**

Emergency closed reduction is feasible for anterior shoulder dislocation combined with humeral surgical neck fracture. Therefore, closed reduction and external fixation should be considered as an optional approach, mainly applicable to elderly patients with reducible Type I or Type II fracture-dislocation classification.

## 1 Introduction

Although shoulder dislocation is a common acute injury, anterior dislocation is the most prevalent type, accounting for approximately 95% of all cases (1, 2). However, anterior shoulder dislocation combined with humeral surgical neck fracture is a rare and severe injury. Mostly caused by external trauma, it can also be observed in cases of epileptic seizures or iatrogenic injuries during shoulder dislocation reduction treatment (3, 4). Since this type of complex injury carries risks such as neurovascular injury and impaired blood supply to the humeral head (5, 6), it can be challenging for early emergency management.

Most anterior shoulder dislocations combined with humeral surgical neck fractures require open reduction and internal fixation, or shoulder arthroplasty; however, the closed reduction is also successful in some cases. On one hand, the emergency closed reduction of shoulder dislocation relieves nerve and vascular compression (7), as well as creates conditions for subsequent internal fixation surgery. On the other hand, closed reduction and effective external fixation can serve as the definitive treatment option, for example, this can be applied to elderly and frail patients.

Current studies have researched the selection of treatment methods, closed reduction techniques, and clinical prognosis of the disease (8–10); however, very few studies have analyzed the feasibility and clinical prognosis evaluation of closed reduction in such cases, i.e., what type of patients exhibit a higher probability of successful closed reduction? How is the shoulder function of patients who underwent closed reduction with external fixation in the later stage of this condition? This is crucial for a precise analysis of the inherent trauma mechanism and the determination of treatment decisions. Therefore, we conducted this retrospective cohort study and evaluated the feasibility of closed reduction for treating elderly cases of anterior shoulder dislocation combined with humeral surgical neck fracture through a fracture-dislocation classification. Compared with the traditional classification of proximal humeral fractures, such as the “Neer classification” (5), this fracture-dislocation classification is more concise and suitable for the classification of anterior shoulder dislocation combined with humeral surgical neck fracture, and can evaluate the reducibility of this disease.

## 2 Materials and methods

### 2.1 Study design

This study is a single center, retrospective cohort study, from January 2010 to March 2024. The purpose is to clarify the feasibility and impact on shoulder joint function of closed reduction and external fixation in elderly patients with anterior shoulder dislocation combined with humeral surgical neck fracture based on a new fracture-dislocation classification.

### 2.2 Diagnosis and fracture-dislocation classification

The diagnostic criteria for anterior shoulder dislocation combined with humeral surgical neck fracture are: (1) A history of trauma; (2) The clinical manifestations of acute anterior shoulder dislocation like shoulder pain, deformity, and limited mobility; (3) x-ray and/or computed tomography (CT) examination can provide a clear diagnosis in such cases as it is a rare and severe injury.

**Figure 1:**
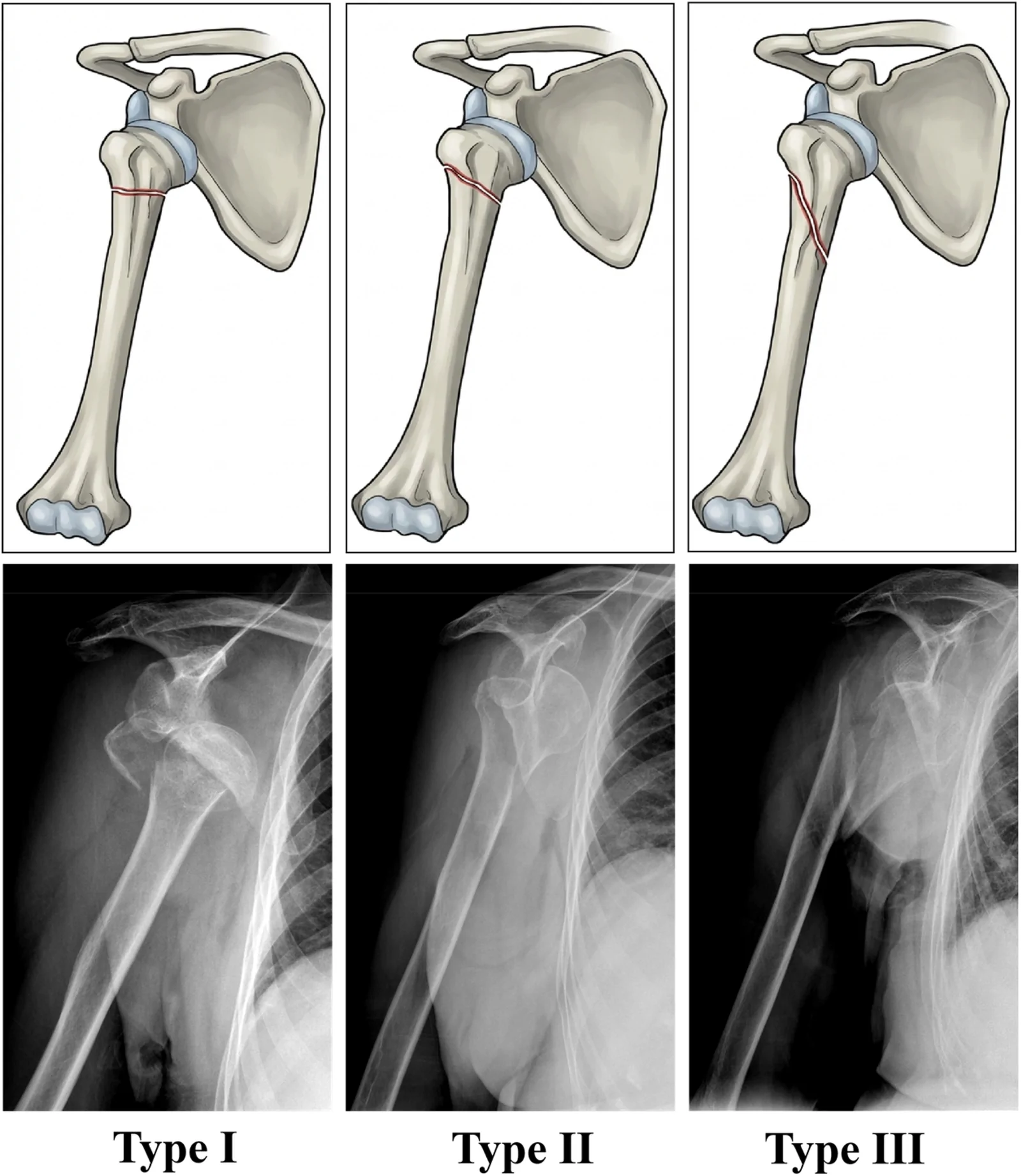
The fracture-dislocation classification of anterior shoulder dislocation combined with humeral surgical neck fracture demonstrates three types. In Type I, the fracture line is transverse to the lower edge of the humeral tuberosity and might be accompanied by fractures of the greater or lesser tuberosity. In Type II, the fracture line is short oblique, running from the top outside to the bottom inside, and in Type III, the long oblique fracture line runs from top to bottom and involves the medial upper segment of the humerus.

According to the morphology of the humeral surgical neck's fracture line, anterior shoulder dislocation combined with humeral surgical neck fracture can be classified into three fracture-dislocation types: Type I, showing transverse humeral surgical neck fracture with anterior shoulder dislocation and accompanied by fractures of the greater or lesser tuberosity of humerus simultaneously; Type II, exhibiting short oblique fracture of the humeral surgical neck accompanied by anterior shoulder dislocation, and Type III, showing long oblique fracture of the humeral surgical neck with anterior shoulder dislocation (Figure 1). For determination of the fracture-dislocation classification, the two trauma surgeons independently viewed the x-ray images and made judgments. In cases of disagreement, a third person was introduced to reach a consensus.

### 2.3 Inclusion and exclusion criteria

The inclusion criteria were: (1) Those who met the diagnostic criteria for anterior shoulder dislocation and humeral surgical neck fracture; (2) Elderly patients, i.e., those aged over 65 years; (3) Patients who attempted closed reduction in the emergency department and had complete imaging data, and (4) Inclusion of retrospective studies, with a follow-up period of more than one year.

The exclusion criteria were: (1) Those with comminuted fractures of the humerus surgical neck that could not be classified into the aforementioned three types; (2) Patients having combined lesions of the affected limb, fractures of other parts of the shoulder, such as scapula and clavicle fractures, rotator cuff injuries, and scapulohumeral periarthritis; (3) Patients with combined brachial plexus nerve and vascular injuries; (4) Patients who underwent surgical treatment due to failed closed reduction, and (5) Other factors, including shoulder trauma, surgery, chronic diseases, etc., had an impact on functional evaluation during the follow-up.

### 2.4 Intervention

All patients underwent treatment in two stages. In phase one, we attempted emergency closed reduction and external fixation with braces under brachial plexus anesthesia, while in phase two, complete imaging examinations were conducted for patients who had successfully undergone emergency reduction. According to the fracture reduction situation, for patients with a reduction grade evaluation of “poor,” open reduction and internal fixation surgery is needed. For patients with a grade evaluation of either “excellent” or “good”, external fixation with braces should be performed. However, open reduction and internal fixation surgery should be performed after completing preoperative routine examinations for patients with failed dislocation reduction.

#### 2.4.1 Closed reduction

According to the previous research, anterior shoulder dislocation should be reduced first, followed by the fracture, when closed reduction should be performed (8). The initial step is to reduce the dislocation. The patient is kept in a supine position, with one assistant fixing the patient's torso and the other assistant performing mild resistance traction by abducting the affected limb. The operator faces the patient, feels the dislocated humeral head in the armpit, and uses the thumb to push the humeral head. The sound of joint reduction indicates a successful reduction. The second step involves reducing the humeral surgical neck fracture. The assistant continues to pull, and the operator squeezes the fractured fragment to reduce it. Finally, the affected limb is adducted and rotated internally, followed by fixing the elbow joint with a brace post-flexion.

#### 2.4.2 Surgical treatment

The surgical procedure was performed by trauma surgeons from the same team. A standardized surgical procedure was adopted (11, 12), involving open reduction combined with PHILOS locking plate fixation for treatment. For patients with severe fracture-dislocations where effective internal fixation is not feasible or there is a high risk of complications (humeral head ischemic necrosis and nonunion of fractures), shoulder arthroplasty was performed.

#### 2.4.3 Post processing

Conservative treatment involved external fixation of the patient for 4–6 weeks, while the affected limb's swelling and blood circulation were observed. After the fixation was released, the patient was guided to perform functional exercise. Postoperative routine treatment for surgical patients included anti-inflammatory and infection prevention measures, as well as guidance on functional exercise. Functional exercise refers to assisted exercise within 2 weeks and active exercise after 2 weeks. It includes activities in four directions of the shoulder joint: lifting, abduction, external rotation, and internal rotation, with a total duration of 8 weeks.

### 2.5 Observation indicators

#### 2.5.1 Imaging evaluation

The patients underwent x-ray examination after closed reduction and surgery to evaluate the quality of fracture reduction. Anatomical reduction was deemed excellent, displacement <3 mm was considered good, and displacement≥3 mm was poor. They were followed up after one year, and the fracture healing status and whether humeral head ischemic necrosis has occurred was evaluated through x-ray examination. It should be noted that x-ray examination is necessary during follow-up, and not all patients have undergone CT examination. During follow-up, when imaging reveals localized increased density of the humeral head, cystic changes, bone collapse, and joint space narrowing, it is considered that humeral head ischemic necrosis has occurred.

#### 2.5.2 Shoulder joint function evaluation

All patients were followed up for more than one year and evaluated for efficacy using the Constant-Murley score (13, 14). During the follow-up period, closely monitor the patient's neurological function and blood circulation in the affected limb. If any neurological or circulatory impairment occurs, emergency treatment is required.

### 2.6 Statistical processing

SPSS 26.0 software (IBM, USA) were used to perform statistical analysis. Measurement data were expressed in the form of mean ± standard deviation, while count data were expressed in the form of the number of cases (%). The shoulder function score and age group comparison were conducted using an independent sample t-test. Fisher's exact probability method helped to compare the fracture classification (types I, II, III), fracture reduction quality (excellent, good, poor), fracture site (left shoulder, right shoulder), and gender (male, female) between groups, respectively. The test level value was considered as 0.05 on both sides.

## 3 Results

### 3.1 General information

**Figure 2:**
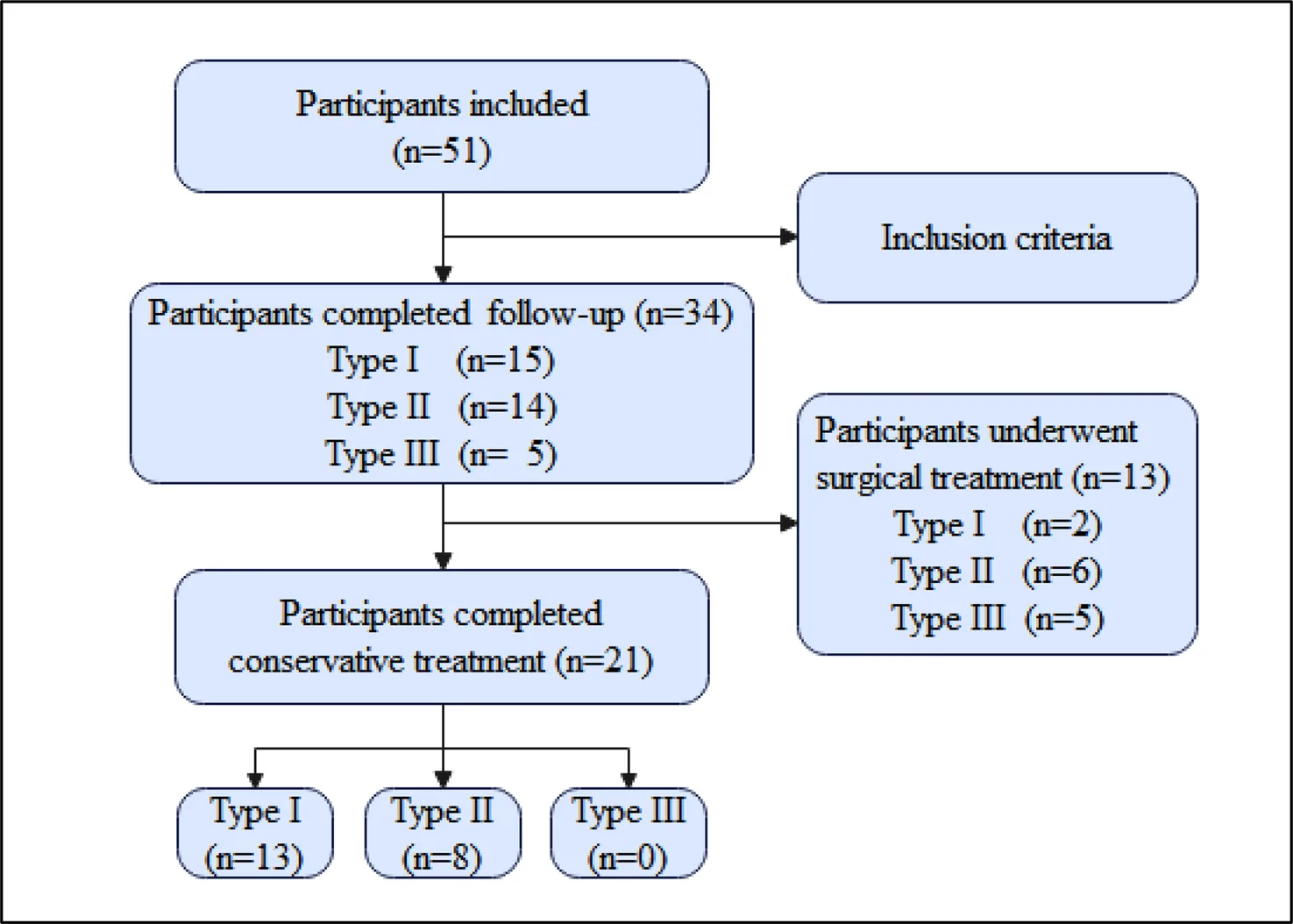
Flowchart of the cohort study.

A total of 51 patients with anterior shoulder dislocation combined with humeral surgical neck fracture from Suzhou TCM Hospital Affiliated to Nanjing University of Chinese Medicine participated in this retrospective cohort study. After screening, 38 patients were included in the cohort, and ultimately 34 patients completed the follow-up (Figure 2). Among them, there were 11 males and 23 females. Their ages ranged from 65 to 90 years, with an average of (74.15 ± 7.36) years. The duration from injury to emergency treatment were 1–24 h, with an average of (6.66 ± 5.55) hours. We included 15 and 19 cases of the left shoulder and the right shoulder, respectively (Table 1).

**Table 1:** General information of patients (*n* = 34).

Characteristic	Number of patients (%)	Age (years)/Duration (hours)
Gender		
Male	11 (32.35)	
Female	23 (67.65)	
Age		74.15 ± 7.36
Course of disease		6.66 ± 5.55
Injury site		
Left shoulder	15 (44.12)	
Right shoulder	19 (55.88)	
Fracture-dislocation classification		
Type I	15 (44.12)	
Type II	14 (41.18)	
Type III	5 (14.70)	
Treatment method		
Conservative treatment	21 (61.76)	
Surgical treatment	13 (38.24)	

### 3.2 Fracture-dislocation classification and closed reduction

**Figure 3:**
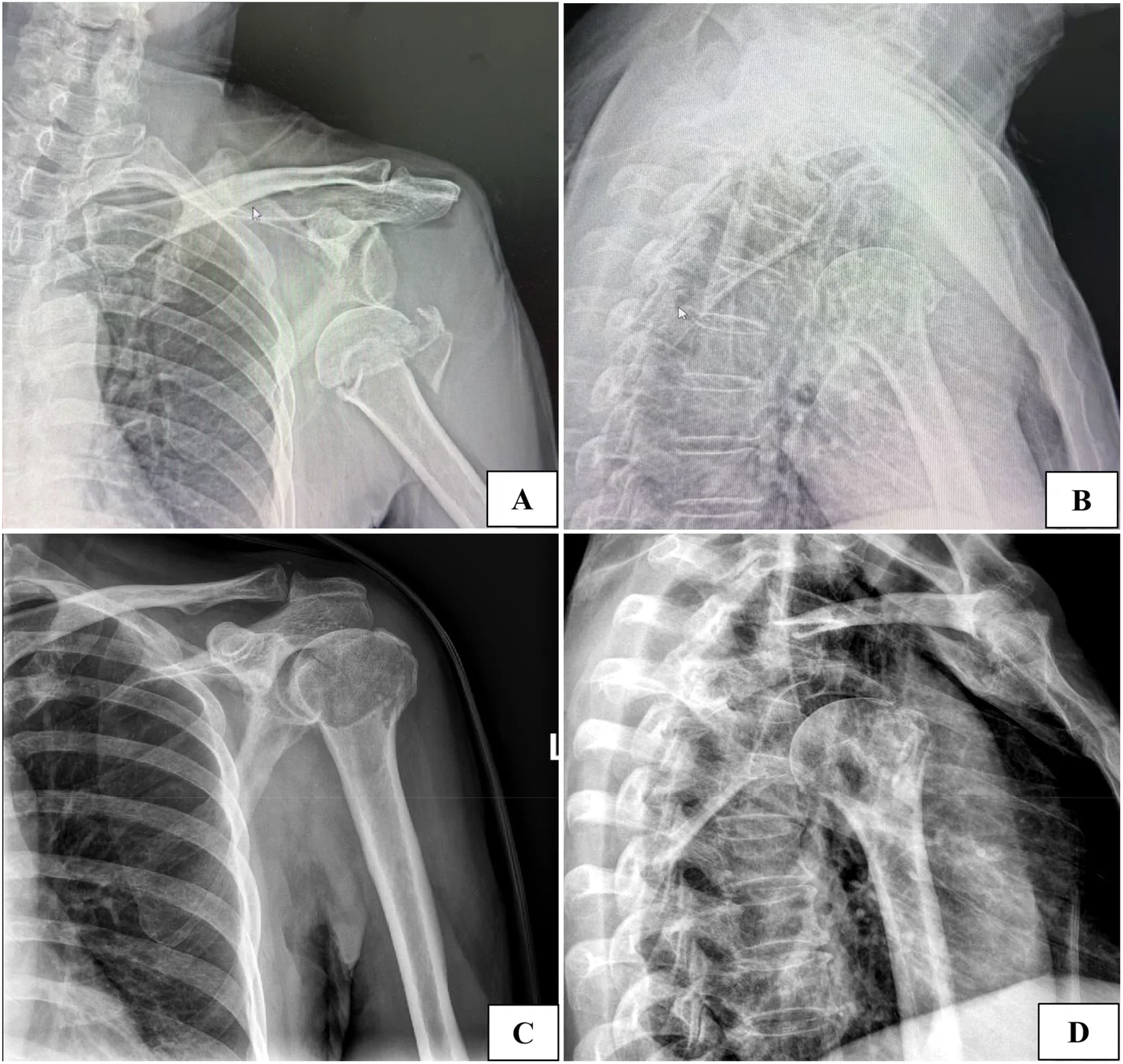
x-ray images from a 69-years-old male with anterior shoulder dislocation combined with humeral surgical neck fracture. **(A,B)** The x-ray shows the fracture line is transverse and belongs to type I fracture-dislocation classification. **(C,D)** After emergency closed reduction and external fixation, x-ray shows the dislocation has been reduced and with a good alignment of the fracture.

All 34 patients comprised 15 cases of type I, 14 cases of type II, and 5 cases of type III, respectively. Notably, 64.71% (22/34) of them achieved successful closed reduction, including 13 cases of type I, 9 cases of type II, and no case of type III. The typical case is shown in Figure 3.

Ultimately, 13 patients received surgical treatment, including 9 cases of open reduction and internal fixation and 4 cases of shoulder arthroplasty. The duration from failed reduction to surgical treatment were 1–7 days, with an average of (3.08 ± 1.66) days. Two type I patients failed closed reduction and underwent surgery. Among type II patients, 5 cases failed closed reduction and underwent surgery, while 1 type II patient exhibited a successful closed reduction but had significant fracture displacement and surgical indications, requiring surgery. Five type III patients had failed closed reduction and underwent surgery (Table 2).

**Table 2:** Closed reduction and surgery of different types of patients.

Classification	Number of cases (*n* = 34)	Success rate of closed reduction (*n* = 22)	Surgical treatment (*n* = 13)	Conservative treatment (*n* = 21)
Type I	15	86.67% (13/15)	13.33% (2/15)	86.67% (13/15)
Type II	14	64.29% (9/14)	42.86% (6/14)	57.14% (8/14)
Type III	5	0% (0/5)	100% (5/5)	0% (0/5)

### 3.3 Clinical efficacy

We followed up 34 patients for 12–18 months, with an average of (14.21 ± 2.01) months. Finally, the Constant Murley shoulder function score for conservative treatment patients ranged from 68 to 95 points, with an average of (83.90 ± 6.62) points.

In the conservative treatment group, there were 13 patients with type I and 8 patients with type II, respectively. The type I group comprised 4 males and 9 females. Their age was (76.23 ± 8.13) years; we considered 7 and 6 cases of left shoulder injury and right shoulder injury, respectively. The type II group included 2 males and 6 females. Their age was (77.38 ± 5.32) years; we considered 4 and 4 cases of left shoulder injury and right shoulder injury, respectively. No difference was observed in baseline data between the two groups, indicating comparability (P1 > 0.999, P2 = 0.728, P3 > 0.999). The imaging results showed that the type I group's quality of fracture reduction was equivalent to that in the type II group (*p* = 0.614). At the last follow-up, the Constant Murley score showed no significant difference between the type I group and the type II group (*P* > 0.05), including no differences observed in pain level, daily life, muscle strength, or activity level between the two groups (Table 3).

**Table 3:** Imaging assessment and follow-up results of shoulder joint function in the two groups (*n* = 21).

Index	Type I group (*n* = 13)	Type II group (*n* = 8)	Inspection value	*P* value
Quality of fracture reduction, *n* (%)				
Excellent	6 (46.15%)	2 (25%)	-	0.614
Good	5 (38.46%)	4 (50%)		
Poor	2 (15.39%)	2 (25%)		
Constant Murley score, point				
Total score	82.69 ± 7.26	85.88 ± 5.25	*t* = −1.074	0.296
Pain level	11.92 ± 3.84	11.88 ± 4.58	*t* = 0.026	0.980
Daily life	16.92 ± 2.47	17.88 ± 1.96	*t* = −0.924	0.367
Muscle strength	23.08 ± 2.53	24.38 ± 1.77	*t* = −1.267	0.221
Activity level (lifting)	6.77 ± 1.30	7.25 ± 1.49	*t* = 0.779	0.445
Activity level (abduction)	6.77 ± 1.54	7.50 ± 1.41	*t* = −1.090	0.289
Activity level (external rotation)	8.46 ± 1.20	8.50 ± 0.93	*t* = −0.077	0.939
Activity level (internal rotation)	8.77 ± 1.54	8.50 ± 0.93	*t* = 0.446	0.661

### 3.4 Complications

At the last follow-up, 8.82% (3/34) and 5.88% (2/34) of patients experienced humeral head ischemic necrosis and nonunion of fractures, respectively. In the conservative treatment group, there were 2 cases of humeral head ischemic necrosis, both classified as type II fracture-dislocation, and 1 case of fracture nonunion, classified as type I. In the surgical treatment group, there was 1 case each of humeral head ischemic necrosis and nonunion of fractures, both classified as type III fracture-dislocation. During the cohort follow-up process, there were no cases of brachial plexus injury or vascular injury occurred.

## 4 Discussion

### 4.1 Classification basis: analysis of fracture-dislocation mechanism

Anterior shoulder dislocation combined with humeral surgical neck fracture is often caused by forced abduction and external rotation. It is generally believed that violence leads to anterior shoulder dislocation first and then causes humeral surgical neck fracture, i.e., fracture is followed by dislocation. However, based on clinical observations, we found that direct assault was the cause of this condition in such patients, and the fracture line was transverse (type I). Thus, we suggest that there is a possibility of dislocation followed by fracture in patients with type I. The transverse fracture line of the humeral surgical neck fracture in type I cases is caused by simple abduction violence. The affected limb is subjected to violence in the abduction and extension positions, thereby resulting in a transverse fracture of the humeral surgical neck. Since the transverse fracture can transmit vertical violence, the additional violence pushes the humeral head out of the glenoid joint and causes anterior dislocation. However, both type II and III patients exhibit oblique fractures and are unable to transmit vertical violence. Apart from abduction violence, they often experience external rotation violence, a fact which aligns with the trauma mechanism of fracture followed by dislocation.

Based on the fracture-dislocation mechanism analysis, we suggest that patients with abduction violence as the primary cause often experience type I fracture-dislocation, and fracture might develop before dislocation. Patients who are subjected to compound violence of abduction and external rotation often experience type II and III fracture-dislocation; in both of these scenarios, fracture is followed by dislocation. The fracture line runs in an oblique direction. Although type III involves the upper segment of the humerus, showing a long oblique line, type II does not involve it, showing a short oblique line.

### 4.2 Feasibility study of closed reduction based on fracture-dislocation classification

This disease is characterized by a fracture accompanied by dislocation, rupture of the shoulder joint capsule, and soft tissue damage. Soft tissue compression is an important obstacle to reduction, especially the obstruction of the short head of the biceps and the coracobrachialis muscle. Therefore, the magnitude of soft tissue damage and obstruction is crucial for evaluating the success rate of closed reduction. The injury mechanism in type I patients involves abduction violence, with fracture followed by dislocation. Since there is a single soft tissue injury pathway, it makes reduction relatively easy. Based on abduction violence history, type II and III patients often experience external rotation violence, which initially leads to the dislocation of humeral head from the weaker part of the joint (front and bottom of the joint) and rupture of the joint capsule (the first soft tissue injury channel). Continued violence results in an oblique fracture and soft tissue injury around it (the second soft tissue injury channel). The compression obstruction of the two soft tissue injury channels is more severe and might be the reason for the challenges in performing closed reduction.

The success rate of closed reduction in this study was 86.67% in type I patients, 64.29% in type II patients, and 0% in type III patients. Type I cases exhibited the highest success rate, followed by type II, while type III cases had the lowest success rate, consistent with the above analysis. Hence, we suggest that this fracture-dislocation classification has significant predictive value for the success rate of the closed reduction.

### 4.3 The significance of closed reduction and the selection of treatment methods

Once a shoulder dislocation occurs, emergency reduction should be performed to avoid prolonged compression-related nerve and vascular damage (15, 16), especially with humeral neck fractures. Proper emergency treatment is crucial for the patient's functional recovery. Adam et al. (17) observed 50 patients with humeral neck fractures accompanied by shoulder dislocation. Among them, 25 patients attempted emergency closed reduction, and finally, 10 patients (40%) had a successful reduction. Thus, we suggest that emergency closed reduction should be attempted for patients with anterior shoulder dislocation combined with humeral surgical neck fracture. On one hand, if the reduction is successful, some patients can avoid surgery and accompanying complications. On the other hand, the successful reduction of the glenohumeral joint preoperatively can reduce soft tissue dissection caused by exposure of the humeral head during surgery and minimize surgery-related blood supply damage (18).

Open reduction and internal fixation or shoulder replacement surgery is the current treatment modality for treating proximal humeral fractures and dislocations (19–21), but for some patients, non-surgical treatment can be attempted, especially for those with surgical contraindications such as advanced age and poor health conditions (22). Bezirgan et al. (23) conducted a follow-up analysis on 45 elderly patients (>60 years) with proximal humeral fractures. The final results showed no significant difference in VAS score, shoulder joint function score, and other aspects between the surgical treatment and the non-surgical treatment groups. Additionally, advancing age can be a crucial factor that leads to the failure of internal fixation in proximal humeral fracture cases (24). In this study, according to the follow-up results, the Constant Murley shoulder function score for conservative treatment patients ranged from 68 to 95 points, with an average of (83.90 ± 6.62) points, and only a small number of patients experienced complications.

The treatment of anterior shoulder dislocation combined with humeral surgical neck fracture should be individualized according to the patient's age, bone quality, degree of fracture displacement and its reducibility, neurovascular status, and functional requirements. Previous studies have reported several treatment strategies for proximal humerus fractures, including two-plate/new plate fixation, emergency evaluation and management, and treatment of humeral fracture nonunion (12, 25–27). In this situation, closed reduction and external fixation should be regarded as an optional choice, mainly suitable for elderly patients with reducible type I or partial type II classification. For patients with type III classification, neurovascular injuries, or irreducible fractures, active surgical treatment should be carried out to avoid missing the treatment opportunity.

## 5 Conclusion

In summary, there is a feasibility of closed reduction for anterior shoulder dislocation combined with humeral surgical neck fracture. This study indicates that closed reduction and external fixation should be considered as an optional approach, mainly applicable to elderly patients with reducible Type I or Type II fracture-dislocation classification. However, the sample size of this study is small, and it was a retrospective design. The results have limitations and need additional confirmation by extensive, prospective studies.

## Data Availability

The raw data supporting the conclusions of this article will be made available by the authors, without undue reservation.
